# Assessing the Reliability of Micro-Doppler Spectrogram Classification from the DIAT-μSAT JPEG Archive

**DOI:** 10.3390/s26195989

**Published:** 2026-09-22

**Authors:** Mert Karahan, Şevki Gani Şanlıöz

**Affiliations:** 1 Department of Electronics and Communication Engineering, Turkish Military Academy, National Defence University, 06420 Ankara, Türkiye; mert.karahan@msu.edu.tr; 2 Faculty of Engineering and Natural Sciences, Istanbul Atlas University, 34408 Istanbul, Türkiye

**Keywords:** radar sensors, micro-Doppler, small aerial targets, DIAT-μSAT, partition sensitivity, archive-order sensitivity, representation randomization, calibration

## Abstract

Reliable AI-assisted interpretation of radar-derived micro-Doppler images requires evaluation protocols that separate image-archive regularities from physical generalization. We audited 4849 DIAT-μSAT JPEG spectrograms and evaluated partition sensitivity, image controls, and representation-randomized training. Raw radar recordings and acquisition metadata were unavailable. A post hoc feature audit found within-class continuity in filename order and a measurable shift between early training and late test blocks in all six classes, using both frozen ResNet18 and HOG features; the source of this order structure remains unknown. Frozen ResNet18 balanced accuracy decreased from 97.71% under a random split to 88.64% under the class-stratified, late-index S3 split. Boundary-restricted images retained class information but did not identify a non-physical artifact or prove shortcut learning. Native JPEG file attributes were separately class-associated but were not supplied to the image classifiers. In a confirmatory long-budget comparison with ten paired optimization seeds and matched checkpoint selection, representation-randomized F2 exceeded the ColorJitter-plus-RandomGrayscale A3WC comparator by 1.69 percentage points in mean seed-wise worst-case balanced accuracy over the protocol-defined K0/K1/K3/K4 rendering set (seed-bootstrap 95% interval +0.45 to +2.78; exact two-sided sign-flip *p* = 0.0352). F2 did not improve the held-out K2 rendering stress test. The results establish an image-level archive-order evaluation stress and a limited finite-rendering training benefit, not acquisition-independent radar recognition.

## 1. Introduction

Radar micro-Doppler signatures encode periodic motion from rotating blades, flapping wings, and other moving target components. These signatures complement bulk Doppler and radar cross-section information when small unmanned aerial vehicles must be distinguished from birds or other low-altitude targets [1,2,3,4,5,6]. Early studies demonstrated target-dependent rotor and wing patterns using spectrograms, cadence representations, bicoherence, and engineered spectral descriptors [1,2,3,4]. Subsequent work extended this foundation through convolutional and recurrent networks, polarimetric processing, data augmentation, and compact models [5,6,7,8,9,10,11,12]. Because these studies used different sensors, class sets, preprocessing chains, and validation units, their headline accuracies do not necessarily measure the same generalization problem.

For a monostatic radar, instantaneous micro-Doppler shift is related to radial micro-motion by fμ(t) = 2v_r_(t)/λ. Rotor radius and angular velocity determine the radial-velocity envelope; blade count and rotor count influence repetition and harmonic structure; and aspect angle projects this motion onto the radar line of sight. Flapping wings produce a different periodic modulation from rigid rotor rotation [1,2,3,4]. These relations provide physical context only: the released JPEGs lack raw samples, calibrated time–frequency axes, and RPM or aspect metadata, so quantitative frequency picking or parameter inversion would not be defensible here.

Accordingly, the convolutional neural network (CNN) is used here as an image classifier, not as a substitute for calibrated spectrum analysis. It can discriminate spatial patterns in rendered pixels, but those patterns may combine micro-Doppler morphology with pseudocolor, axes, interpolation, or compression cues. Classification accuracy therefore does not establish recovery of blade rate, radial velocity, or another physical parameter; such convergence must be tested against raw complex radar samples and acquisition metadata.

A spectrogram is not a neutral observation once it has been exported as a rendered image. Axis layout, source resolution, interpolation, color mapping, compression, and cropping can become statistically associated with class labels. A model may therefore exploit image-production regularities in addition to, or instead of, target-related micro-Doppler structure. High-capacity models can learn such predictive shortcuts within a benchmark [13], while leakage or unmodeled dependence between nominally separate samples can further inflate performance [14]. Validation groups should follow the true dependence unit whenever that unit is known [15]. More broadly, dataset-bias and distribution-shift studies show that within-dataset accuracy need not transfer to separately collected image sets or modestly changed collection conditions [16,17,18].

DIAT-μSAT provides 4849 published JPEG spectrograms from six small-aerial-target categories and has supported a growing line of work on transfer learning, lightweight CNNs, engineered spectral features, recurrent models, complex-valued networks, attention, binarization, and YOLO-derived classifiers [19,20,21,22,23,24,25,26,27,28,29,30]. The original study reported 95% and 97% accuracy for VGG16 and VGG19 under a random 80/10/10 image split [19], while DIAT-RadSATNet emphasized model size and efficiency on held-out images from the same data source [21]. Later studies changed both representation and evaluation: R-PCA with SVM used range- and micro-Doppler images [22]; GRU models used temporal signals and, separately, synthetic additive white Gaussian noise [23,24]; optimized SVMs used engineered spectral features [25]; and complex-valued networks used frequency-domain inputs [26,27]. The attentive binarized CNN study also reported results on RRN_ATR_NET [29], providing a second-dataset check for that architecture.

These results are useful within their stated protocols, but they cannot be ranked solely by headline accuracy because the class subsets, input forms, sampling units, and test definitions differ. The DIAT-specific studies reviewed here do not jointly examine exact duplicates, filename-order sensitivity, boundary-restricted information, native file attributes, class-associated source resolution, matched checkpoint selection, and optimization uncertainty within one auditable protocol. In addition, the released JPEG archive provides no verified session, target-instance, range, aspect-angle, or rotational-speed grouping variables. Filename blocks can therefore serve only as diagnostic sensitivity controls, not as acquisition batches or external domains. This leaves a methodological gap between achieving high accuracy within the archive and establishing which conclusions are robust to plausible archive-level evaluation choices.

In this study, reliability is used in a deliberately bounded sense: the stability of conclusions within the released JPEG archive under alternative partitioning, image rendering, source-resolution, checkpoint-selection, and optimization conditions. It does not denote generalization across acquisition sessions, sensors, sites, target populations, or physical operating conditions. Raw radar recordings, physical amplitude and phase information, and the metadata needed to define those external conditions are unavailable; consequently, the study cannot infer physical signal-to-noise ratio, RPM, aspect angle, target identity, or acquisition-session independence.

Against this background, the central research question is as follows: within the released DIAT-μSAT JPEG archive, which conclusions about micro-Doppler classification remain after controlling for duplicate and filename-order structure, image-production factors, source-image resolution, checkpoint selection, and training variability? The analysis addresses two linked components of this question. First, it determines how strongly reported performance depends on the partition and on archive-associated image or metadata controls. Second, it tests whether representation-randomized training improves a protocol-defined finite-rendering worst-case endpoint relative to a matched ColorJitter-plus-RandomGrayscale policy under the same long-training and checkpoint-selection protocol.

The study makes two bounded contributions. First, it identifies measurable feature-space structure along filename order and uses it to characterize S3 as an archive-order extrapolation stress, while retaining duplicate, boundary, metadata, and source-resolution controls to delimit interpretation. This is a post hoc image-level finding, not evidence that file indices encode acquisition sessions or physical conditions. Second, it compares representation-randomized training with a matched augmentation policy using ten paired optimization seeds, a protocol-defined finite-rendering endpoint, and separate uncertainty analyses. The contribution is a reproducible account of which claims this JPEG archive supports, not a new maximum-accuracy classifier or a physical-domain robustness claim.

## 2. Materials and Methods

The workflow first audits the released archive, then fixes the partition and image-control protocols, trains the specified baselines and ResNet18 policies, selects checkpoints only on validation data, and finally evaluates held-out predictions with paired uncertainty analyses (Figure 1). No k-fold cross-validation was used. S0–S3 are fixed train/validation/test assignments; the alternative S3 block lengths and offsets are protocol-sensitivity analyses rather than folds of one cross-validation procedure.

### 2.1. Dataset Scope

DIAT-μSAT contains spectrograms derived from small-aerial-target micro-Doppler measurements collected with a 10 GHz X-band continuous-wave radar [19,20]. The available archive contains no raw .mat recordings; it comprises 4849 JPEG images in six categories: two-blade rotor, three-short-blade rotor, three-long-blade rotor, quadcopter, bionic bird, and a scene in which a bird and a two-blade rotor are present simultaneously. The sixth category is therefore an observation of two concurrent targets, not one composite object.

For every file, the audit recorded a canonical label, image dimensions, native file size, SHA-256 digest, and perceptual similarity descriptors. All 800 bionic-bird images are 700 × 525 pixels, whereas the other 4049 images are 1400 × 1050 pixels. Two exact duplicate pairs were found, and the three-long-blade-rotor filename sequence contains one missing index. These findings motivated explicit controls for class-associated rendering and filename order.

Exact duplicates were identified by identical SHA-256 digests. For the near-duplicate audit, a central analysis crop spanning 12–84% of image width and 10–88% of image height was converted to grayscale. A 64-bit perceptual hash (pHash) was computed from the thresholded 8 × 8 low-frequency discrete cosine transform (DCT) coefficients of a 32 × 32 thumbnail, excluding the DC coefficient from the median; a 64-bit difference hash (dHash) was computed from a 9 × 8 thumbnail. Nonidentical pairs were grouped only when pHash Hamming distance was ≤6, dHash Hamming distance was ≤18, and global structural similarity index (SSIM) was ≥0.99. Accepted pairwise links were merged transitively. These thresholds were fixed before downstream model evaluation. No nonidentical pair satisfied all three criteria, so S2 added no group beyond the two exact-duplicate pairs. The principal archive-audit findings are summarized in Table 1.

The visual examples in Figure 2 show class-dependent image morphology such as differences in band extent, ridge repetition, and diffuse versus compact energy patterns. Such appearances can be physically consistent with differences in rotor/flapping dynamics, blade geometry, rotor count, and aspect. They are treated only as qualitative context because the JPEG archive does not support calibrated frequency, RPM, or construction-parameter estimates from these panels.

### 2.2. Fixed Data Partitions

S0 is a random 80/10/10-style split retained only for secondary comparison with prior work. S1 keeps exact duplicates in the same partition. S2 applies the perceptual-similarity rule fixed before downstream model evaluation, but no nonidentical near-duplicate group met the threshold; consequently, all 4849 S1 and S2 assignments are identical. S2 is retained only to document the negative near-duplicate audit and is not treated as independent evidence beyond S1. The main S3 diagnostic partition sorts filenames within each class and allocates contiguous blocks. A sensitivity analysis used block lengths 10, 20, 40, and 80 and L20 offsets 0, 5, 10, and 15. S3 is a filename-order sensitivity control, not a measurement-session, target-identity, range, aspect-angle, or RPM-independent partition; full results are provided in Supplementary Table S1 and Figure S1.

To determine whether S3 follows any observable image structure, we performed a post hoc, class-conditional audit using the cached I2 HOG and ImageNet-frozen ResNet18 features. Each feature vector was L2-normalized. Within each class, mean cosine similarity of consecutive file indices was compared with 499 random permutations of that class’s index order. We also compared the Euclidean distance between the mean feature vectors of the S3 training and late-index test sets with 499 random assignments preserving their class-specific sizes; validation images were omitted from this distance. One-sided empirical *p*-values were used: (1 + number of null statistics at least as large as observed)/500. As sensitivity checks, both tests were repeated on grayscale I3 features after removing one image from each of the two exact-duplicate pairs. These exploratory, multiple class-feature comparisons were not prespecified as confirmatory endpoints and cannot identify an acquisition mechanism. Full values are in Supplementary Table S8. The fixed partition counts and roles are summarized in Table 2.

### 2.3. Input and Control Families

ROI extraction was defined by a deterministic image-domain rendering-boundary rule rather than by manual per-image selection. A pixel was treated as content when its mean RGB value was below 245; rows and columns were retained when the fraction of content pixels was strictly greater than 0.25. The bounding box spanned the first through last retained row and column, using left/top-inclusive and right/bottom-exclusive coordinates. Applied to all 4849 images, the rule yielded (91, 39, 633, 467) for all 800 images of 700 × 525 pixels. Among the 4049 images of 1400 × 1050 pixels, the modal box was (181, 78, 1268, 935) for 3861 images; 184 differed only at the top boundary by one pixel, and four differed only at the right boundary by one pixel. To avoid adaptive per-image cropping, the two source-size-specific modal boxes were fixed for all downstream variants, with a normalized fallback for unobserved dimensions. Eighteen deterministic examples (the first, middle, and last figure number in each class) were visually checked against the detected and applied boxes. This procedure identifies the colored spectrogram rendering boundary in the released JPEGs; it is not physical radar-signal segmentation.

All model inputs were mapped to a common 224 × 224 space. I0 denotes the original full RGB image; I1 the commonly recoded full image; I2 the RGB spectrogram ROI without the surrounding white canvas; I3 the grayscale ROI; I4 a fixed contrast or color remapping; I5 a boundary-residual diagnostic after masking the central spectrogram region; and I6 a separate metadata-only archive diagnostic. I2 was selected as the principal input because it removes the outer white canvas while preserving the performance range of I0.

To isolate the class-associated source-image dimensions, we defined an additional sensitivity input, I2C. The same ROI coordinates used for I2 were applied first. ROIs from the 1400 × 1050 source images (1087 × 857 pixels) were resampled to 542 × 428 pixels, matching the ROI size obtained from the 700 × 525 bionic-bird images, before the common Lanczos letterboxing step to 224 × 224. All other image encoding, S3 split identities, feature extraction, and validation-based classifier-selection rules were held fixed. I2C equalizes intermediate rendering dimensions only; it does not equalize physical time–frequency resolution or acquisition conditions. The corresponding configurations are summarized in Table 3.

### 2.4. Baseline Models and Uncertainty Analysis

The histogram of oriented gradients (HOG)-SVM baseline used nine orientations, 16 × 16-pixel cells, 2 × 2-cell blocks, L2-Hys normalization, and a class-balanced linear support vector machine (SVM) [31]. The deep-feature baseline used an ImageNet-pretrained ResNet18 encoder with frozen weights; 512-dimensional features were classified by a class-balanced linear SVM [32]. ResNet18 was the only fully fine-tuned deep backbone. HOG-SVM supplies a distinct feature-family control for the archive audit, but it is not a second deep-architecture replication; no claim of architectural generalization is made.

The torchvision implementation followed the standard ResNet18 BasicBlock stage depths (2, 2, 2, 2) and channel widths (64, 128, 256, 512), followed by adaptive global average pooling [32]. For the six-class task, the original 1000-output ImageNet fully connected layer was replaced by a linear classifier with 512 inputs and six outputs; the stem, residual stages, shortcut connections, and block structure were not modified. The frozen-feature model used the 512-dimensional pooled representation, whereas full fine-tuning updated the entire backbone and the new classifier from the first epoch.

The initial policy screen used five fixed optimization seeds, whereas the confirmatory long-budget F2–A3WC comparison used ten paired seeds (2026–2035). Bootstrap resampling was used because the primary effect is a mean of paired computational repeats and because the fixed S3 result may depend on its observed filename-block composition [33]. For the primary seed interval, each of 10,000 replicates sampled ten seed indices with replacement, recomputed the mean paired F2-minus-A3WC difference, and used the 2.5th and 97.5th percentiles. For the complementary block interval, filename blocks were sampled with replacement separately within each class; the same sampled blocks were applied jointly to both models, all ten seeds, and K0/K1/K3/K4 before recomputing the mean difference. These intervals quantify optimization variability and conditional sensitivity to the observed filename blocks, respectively; neither represents independent radar measurement campaigns.

### 2.5. Representation Randomization and Calibration

In the initial five-seed full fine-tuning screen, F0 used only the K0 RGB input, F1 used only the K1 grayscale input, and F2 used deterministic training-time randomization among K0, K1, K3, and K4. Standard comparators were A2 RandomGrayscale and A3 ColorJitter plus RandomGrayscale on the same S3 split and seeds. For context, augmentation-based robustness methods such as AugMix have been proposed to improve robustness and uncertainty [34]; AugMix itself was not used in this study. A3WC repeated the A3 training policy but used the same worst-case K0/K1/K3/K4 checkpoint-selection objective as F2. K0 preserves the published RGB ROI, K1 removes pseudocolor by using grayscale, K3 applies a fixed alternative palette, and K4 applies a fixed red–blue channel permutation. These deterministic rendering controls alter color representation while preserving image geometry. K2 additionally applies per-image histogram equalization and was therefore treated as a stronger image-specific synthetic stress test; it was excluded from training and checkpoint selection.

The confirmatory experiment was restricted to F2 and A3WC, preserved the I2 images and S3 identities, and extended both policies to ten paired seeds under a common long-training budget. The rendering set, paired seeds, checkpoint-selection rule, primary endpoint, and 2-point K0 reporting threshold were fixed in a revision-specific protocol before the new long-budget runs. This protocol was not a preregistration preceding access to the historical benchmark results. K0–K4 are image-rendering controls derived from the released JPEGs; they are not radar perturbations, acquisition domains, or physically parameterized SNR, aspect, range, or RPM changes. For each seed, the primary endpoint is the minimum balanced accuracy over K = {K0, K1, K3, K4}; the reported score is the mean of the ten seed-wise minima. K2 and representation-specific results are secondary and descriptive.

For analytical clarity, let *C* denote the number of classes, *m* the model, *s* the optimization seed, and *k* the rendering. The per-rendering balanced accuracy, finite-set checkpoint endpoint, and paired policy effect were defined as follows:

$$BA_{m,s,k}=\frac{1}{C}\sum_{c=1}^{C}\frac{TP_{c}}{TP_{c}+FN_{c}}$$

$$W_{m,s}=\underset{k\in K_{\mathrm{allow}}}{\min}BA_{m,s,k},\;\;\;\;K_{allow}=\left\{K0,K1,K3,K4\right\}$$

$$\Delta_{s}=W_{F2,s}-W_{A3WC,s},\;\;\;\;\bar{\Delta }=\frac{1}{S}\sum_{s=1}^{S}\Delta_{s}$$

Only the mean paired effect over *S* = 10 computational seeds was treated as the confirmatory primary comparison. These expressions define the statistical estimands; they are not a physical radar-propagation model.

### 2.6. Implementation Details

All deep models used torchvision 0.28.0+cpu ResNet18 initialized from locally stored ImageNet weights. The torchvision software is available under the BSD 3-Clause license; neither its source code nor the pretrained weights are redistributed in the accompanying package. The 224 × 224 RGB tensors were normalized with ImageNet channel means (0.485, 0.456, 0.406) and standard deviations (0.229, 0.224, 0.225). The backbone and classifier were trainable from the first epoch. Training minimized class-weighted cross-entropy, with inverse training frequencies normalized to mean one. AdamW used a learning rate of 1 × 10^−4^ and weight decay of 1 × 10^−4^; batch size was 64. The initial five-seed policy screen used a six-epoch ceiling, cosine annealing to 1 × 10^−6^, and early stopping after epoch 3 with patience 2. Because this ceiling was frequently reached, the confirmatory F2-A3WC experiment used the same optimizer and loss but a 30-epoch ceiling, a minimum of 10 epochs, patience 5, and CosineAnnealingLR with T_max = 30. It used ten paired seeds (2026–2035), CPU execution, eight computational threads, and no additional geometric augmentation.

For a sample with one-hot target *y* and predicted class probabilities *p*, the class-weighted training loss was

$$L_{CE}=-\sum_{c=1}^{C}\alpha_{c}y_{c}\log\left(p_{c}\right),\;\;\;\;\alpha_{c}\propto \frac{1}{n_{c}},\;\;\;\frac{1}{C}\sum_{c=1}^{C}\alpha_{c}=1$$

Here, *n_c_* is the training count for class *c*; the inverse-frequency class weights were normalized to have a mean of one.

F0 and F1 checkpoints maximized balanced accuracy on their matched validation representation. F2 and A3WC checkpoints maximized the minimum validation balanced accuracy across K0, K1, K3, and K4; test data were never used for checkpoint selection. For F2, every sample received exactly one of the four training representations per epoch by SHA-256 (sample_id|epoch|seed|representation) modulo four, giving each representation nominal probability 0.25 without hidden random-number state. A2 converted an input to grayscale with probability 0.25. A3 and A3WC combined that operation with brightness, contrast, and saturation factors sampled from [0.8, 1.2] and hue from [−0.05, 0.05], applied in a deterministic hashed order. In the confirmatory comparison, initialization, classifier initialization, and sample-order policy were paired within each seed. No geometric augmentation was used.

For calibration, U0 denotes the uncalibrated logits and C1 a single scalar temperature fitted by bounded minimization of multiclass negative log-likelihood (NLL) on pooled S3 validation logits from K0, K1, K3, and K4 [35]. K2 was excluded from fitting. Expected calibration error (ECE) used 15 equal-width maximum-probability bins, and the multiclass Brier score was also reported. Both U0 and C1 test results were retained. A calibration change was described as beneficial only if C1 reduced allowed-set mean NLL, did not increase allowed-set mean ECE, and did not increase K0 NLL by more than 0.02. This reporting rule did not select a network checkpoint or suppress any test result.

## 3. Results

### 3.1. Random-to-Blocked Partition Sensitivity

Frozen ResNet18-I2 balanced accuracy was 97.71% on random S0, 96.67% on the single duplicate-aware S1/S2 result, and 88.64% on the main S3 filename-order sensitivity partition. Because S1 and S2 produced identical assignments and predictions, they are not counted as separate corroborating experiments. The S0-S1/S2 difference was 1.04 percentage points, whereas the S1/S2-S3 difference was 8.03 points. Across the seven predeclared S3 block variants, frozen-ResNet18 balanced accuracy remained between 88.64% and 90.10%; HOG-SVM remained between 79.39% and 81.68% (Supplementary Table S1). The random-to-blocked reduction therefore did not depend on one block length or L20 grid origin. With full F0 fine-tuning, S0 yielded 98.92 ± 0.45% and S3 88.97 ± 2.13%; S0 was higher for all five seeds. The mean paired gap was 9.94 ± 2.19 points, but the exact two-sided sign-flip *p*-value was 0.0625, so no *p* < 0.05 claim is made. This is evidence of sensitivity to the imposed filename-order partition, not proof of acquisition leakage, non-i.i.d. sampling, or physical sample dependence. The complete frozen-feature partition chain is reported in Table 4.

### 3.2. Observable Structure in Filename Order

The post hoc feature audit found that consecutive file indices were more similar than randomly permuted orders in every class. The adjacent-cosine excess ranged from 0.01975 to 0.07504 for frozen ResNet18-I2 and from 0.00456 to 0.06919 for HOG-I2. The S3 early-training versus late-test centroid distance was 2.25–4.87 times the mean size-matched random-assignment distance for ResNet18-I2 and 1.56–4.53 times for HOG-I2. Every class-feature comparison exceeded all 499 permutation null values for both statistics (empirical one-sided *p* = 0.002, the permutation resolution). Grayscale I3 analyses excluding the two duplicate second members showed the same direction in all classes (Supplementary Table S8). Thus, filename order carries observable image-feature structure; neither these features nor the file indices identify the physical source of that structure. S3 tests extrapolation to late archive indices, not independence across sessions, targets, or operating conditions.

### 3.3. Boundary Controls as Non-Identifying Diagnostics

The original I5-B0 control produced 67.43% balanced accuracy with HOG-SVM and 69.54% with ResNet18. Expanding the mask by two, four, and eight pixels reduced performance to 59.90%, 33.33%, and 16.67% for HOG and to 53.93%, 33.33%, and 16.67% for ResNet18. At B4, only bionic-bird images retained non-white pixels: the mean remaining proportion was 0.2267%, all 800 bionic-bird images were non-white, and all 4049 images from the other five classes were completely white. The classifiers produced identical B4 predictions. The 33.33% result therefore reflects separation of the bird-specific residual from uniform white inputs. B8 was completely white and reached the six-class chance level. B4 and B8 validate the construction of the increasingly strict null inputs; B0 and B2 can contain both genuine perimeter time-frequency content and rendering traces and therefore do not, by themselves, prove shortcut learning. Figure 3 illustrates the progressive boundary-masking diagnostic and the associated performance trend.

### 3.4. Metadata-Only Archive Diagnostic

No file-level attribute was supplied to HOG-SVM or ResNet18; I6 was a separate metadata-only decision-tree diagnostic. The I6 tree was decomposed into four controlled feature groups. Native file size alone reproduced the 44.38% balanced accuracy of the complete metadata model and accounted for all feature importance in that tree. Width plus height yielded 33.33%, whereas the JPEG coding fields yielded 16.67%. The result supports a narrow conclusion: native JPEG file size has a class-associated dataset-level relationship within the released archive, not that either image classifier read file metadata. File size jointly reflects image content, compression, and the rendering pipeline and therefore does not identify a single causal mechanism. The controlled metadata ablation is summarized in Table 5.

### 3.5. I2 Inner-Edge and Rendering Experiments

The additional control requested for boundary-restricted learning was performed on the fixed S3 identities. Suppressing 2, 4, and 8 pixels along the inner content boundary of I2 produced HOG-SVM balanced accuracy of 79.50%, 77.85%, and 82.33% and ResNet18 balanced accuracy of 86.97%, 88.63%, and 90.76%, compared with 81.18% and 88.64% for C0, respectively. Conversely, training and testing on isolated boundary rings R2, R4, and R8 produced HOG-SVM balanced accuracy of 67.12%, 68.44%, and 73.74% and ResNet18 balanced accuracy of 75.03%, 78.61%, and 81.97%, all above the six-class chance level of 16.67% (Supplementary Table S6). The absence of a monotonic and model-consistent decline under inner-boundary suppression shows that I2 performance cannot be explained solely by a few boundary pixels. The ring results demonstrate that boundary-restricted inputs retain class-related information, but the rings also retain the perimeter of genuine time–frequency content. They therefore cannot identify that information as a non-physical plotting artifact.

The common-intermediate-resolution control did not produce a material or model-consistent performance change under the fixed S3 test identities. For frozen ResNet18 features with a validation-selected LinearSVC, balanced accuracy was 88.64% for both I2 and I2C (I2C-I2: 0.00 percentage points; paired class-stratified filename-block bootstrap 95% interval: −0.42 to +0.42). For HOG16 features, balanced accuracy changed from 81.18% to 81.78% (+0.60 points; 95% interval: −0.85 to +2.07). Bionic-bird recall remained 100% for frozen ResNet18 and changed from 97.50% to 98.75% for HOG. These changes were below the protocol-defined decision threshold for a long fine-tuning rerun.

To determine whether the random-to-blocked reduction was driven by the single source-resolution-confounded category, we repeated the frozen-feature I2 analysis after excluding all 800 bionic-bird images while preserving the original S0 and S3 memberships. The retained five-class dataset contained 4049 images (S0 test *n* = 404; S3 test *n* = 390). LinearSVC hyperparameters were reselected using only the filtered validation partitions, after which the classifier heads were refitted on the corresponding train-plus-validation sets. Frozen ResNet18 balanced accuracy was 97.25% on S0 and 86.12% on S3, a gap of 11.13 percentage points (independent class-stratified test-sample bootstrap 95% interval: 7.76 to 14.87). HOG-SVM yielded 83.30% and 78.18%, respectively, a gap of 5.12 points (95% interval: −0.16 to 10.33; Supplementary Table S7). Thus, the partition sensitivity was not eliminated by removing the only category with a distinct source resolution. Because S0 and S3 contain different test identities, these intervals quantify diagnostic split sensitivity rather than a paired causal effect.

K0 is the published RGB ROI; K1 is grayscale; K2 is per-image histogram-equalized grayscale; K3 uses a fixed alternative palette; and K4 swaps the red and blue channels. When models were retrained in each representation, ResNet18 maintained 89.33% on K3 and 87.85% on K4, close to 88.64% on K0. In contrast, a model trained only on K0 fell to 36.62% on K1, 40.50% on K2, 40.03% on K3, and 59.62% on K4. The issue is not that alternative palettes are unlearnable; rather, a decision boundary fitted to a single palette fails under a rendering shift.

### 3.6. Confirmatory Training-Policy Comparison

K2 was not used in any training policy or checkpoint selection. In the initial six-epoch, five-seed screen, F2 exceeded A3WC by 4.57 points in mean allowed-set worst-case balanced accuracy. The confirmatory common long-budget comparison reduced this difference to 1.32 points for the same 2026–2030 seeds and to 1.69 points across all ten paired seeds. Long-budget means were 89.94 ± 1.57% for F2 and 88.25 ± 1.28% for A3WC. The paired-difference standard deviation was 1.98 points; the seed-bootstrap 95% interval was +0.45 to +2.78 points, and the exact two-sided sign-flip *p*-value was 0.0352. F2 was higher in seven seed pairs and lower in three. Its mean K0 difference was −0.26 points (seed-bootstrap 95% interval: −1.55 to +0.79), which did not exceed the protocol-defined 2-point material-loss threshold; this threshold is a reporting rule, not a formal non-inferiority margin. The shorter and longer protocols used different cosine and early-stopping budgets, so their difference cannot be attributed to epoch count alone. The full ten-seed comparison is reported in Table 6.

### 3.7. Group-Aware Uncertainty Analysis

For the confirmatory long-budget comparison, resampling the 24 observed filename blocks within class 10,000 times produced a conditional 95% interval of +0.74 to +3.56 points for the F2-A3WC allowed-set worst-case difference. This complements, but does not replace, the +0.45 to +2.78-point seed-bootstrap interval: the former varies the observed filename blocks while holding the ten trained seed pairs fixed, whereas the latter characterizes optimization variability on the fixed test set. The exact sign-flip *p* = 0.0352 applies only to the ten paired computational repeats and assumes sign symmetry under the null. There was one protocol-defined primary comparison and endpoint; representation-specific scores, K2, class recalls, and epoch summaries were secondary descriptions without a secondary *p*-value search. Filename blocks are not verified radar sessions. Seed-wise results and the compact uncertainty summary are provided in Supplementary Tables S2 and S3.

### 3.8. Calibration and Reliability

For F0, C1 increased NLL from 0.6289 to 0.6412, ECE from 0.0714 to 0.0788, and Brier score from 0.3170 to 0.3207; it did not improve calibration under the predefined descriptive criteria. For F2, C1 reduced NLL from 0.3210 to 0.3009, ECE from 0.0578 to 0.0496, and Brier score from 0.1592 to 0.1553 over the allowed representations. The seed-paired bootstrap 95% interval for the F2 C1-U0 NLL difference was [−0.0347, −0.0055]. K2 was excluded from temperature fitting, yet F2 NLL on K2 decreased from 1.4803 to 1.3567. Temperature scaling did not alter any predicted class label. The full calibration summary is provided in Supplementary Table S4.

### 3.9. Class-Wise Failure Structure

In the confirmatory long-budget results, F2’s clearest K0 weakness remained the three-short-blade-rotor class, with 73.25% mean recall; the corresponding A3WC recall was 73.63%. Under the held-out K2 stress test, F2 recall fell to 0.53% for quadcopter and 22.75% for the three-long-blade rotor, compared with 10.00% and 34.50% for A3WC. F2 therefore did not provide a general advantage on K2. Bionic-bird recall remained 100% for both models, while the simultaneous bird-rotor scene reached 93.87% for F2 and 97.20% for A3WC. These class-specific failures are secondary descriptive results and show why the aggregate finite-set metric should not be interpreted as general robustness. Figure 4 shows the corresponding mean row-normalized F2 confusion matrices for K0 and K2.

## 4. Protocol-Aware Comparison with Published DIAT-μSAT Studies

Published DIAT-μSAT values do not answer one experimental question. Studies differ in input domain, preprocessing, class subset, sampling unit, validation design, and use of a second dataset. Table 7 records these protocol dimensions explicitly rather than ranking methods by headline accuracy. In particular, an image-level random or internally held-out test does not establish independence from acquisition batches, and a synthetic corruption does not constitute external sensor validation. The lower S3 K0 balanced accuracy, compared to the commonly reported 97–99% internal values, is therefore interpreted as partition sensitivity within the released archive.

## 5. Discussion

The principal result is an empirically bounded evaluation of the released JPEG archive. Filename order contains measurable within-class image-feature structure, and the late S3 blocks differ from earlier blocks in two feature families. The random-to-S3 accuracy gap is consistent with an archive-order extrapolation stress, although the two protocols also differ in local-block separation. It does not quantify session-independent or physical radar generalization. Boundary controls remain non-identifying diagnostics rather than evidence of a specific non-physical shortcut. The matched long-budget training comparison adds a separate, limited benefit over a finite rendering set. These results change how within-archive accuracy should be interpreted but do not validate a new sensor-domain classifier.

### 5.1. Principal Scientific Contribution

The positive image-level finding is that filename order is not visually arbitrary: within each class, adjacent images are more similar than permuted neighbors, and the held-out late block is more separated from early training images than a size-matched random allocation. This gives S3 a defined role as an archive-order extrapolation stress. It does not reveal why the ordering exists, whether neighboring files share an acquisition session, or how a model would perform on new physical measurements. The second contribution is a matched long-budget comparison of two training policies on a finite rendering set, with the K2 failure kept explicit. The methodological value lies in linking each evaluation result to the limited claim it can actually support. For similar provenance-poor image archives with ordered filenames, we recommend reporting a class-conditional order diagnostic and an order-sensitive holdout alongside random-split accuracy, while avoiding a physical-domain interpretation of that holdout.

### 5.2. Interpreting the Random-Split Gap

The S0–S3 gap persisted for both frozen and fully fine-tuned ResNet18, while S1 and S2 are one duplicate-aware result rather than independent confirmations. The new feature audit shows that file index is associated with image content across all six classes, including after grayscale conversion and removal of the two exact-duplicate second members. Accordingly, S3 is not merely an arbitrary shuffle of filenames: its late-index test set probes a measured archive-order shift. At the same time, S0 and S3 differ in both local-block separation and early-to-late placement, so their accuracy difference cannot be causally assigned to one mechanism. Because the index has no verified acquisition meaning, neither the feature audit nor the accuracy gap establishes session leakage or generalization to independent radar measurements.

### 5.3. Scope of the Artifact Evidence

The separate I6 metadata-only decision tree reproduced 44.38% balanced accuracy using native file size alone, with all importance assigned to file_bytes. No file-level attribute was supplied to HOG-SVM or ResNet18. Width plus height exposed the distinct bionic-bird source resolution and reached 33.33%, whereas JPEG sampling, progressive, EXIF, and ICC fields remained at chance. File size is a composite trace of content, compression, and rendering, so this dataset-level association does not identify a single production mechanism. The B4 and B8 experiments establish increasingly strict image-domain null inputs, while the boundary-ring results show that the retained perimeter remains class-informative without distinguishing genuine time–frequency content from rendering traces.

Although source-image dimensions perfectly distinguish the bionic-bird category from the other categories in the released archive, the I2C control indicates that this association has little residual influence after ROI extraction and final 224 × 224 mapping in the two evaluated feature pipelines. The conclusion is deliberately narrow: this control does not prove the absence of resolution shortcuts in every trained network, and it does not convert the dataset into an externally validated benchmark.

The five-class exclusion control further narrows the resolution-based alternative explanation: removing every bionic-bird image did not attenuate the S0–S3 reduction in frozen ResNet18 performance, and HOG-SVM changed in the same direction. This does not identify the dependence mechanism within the remaining classes, but it shows that the main filename-order sensitivity finding is not solely a consequence of the one class with different native image dimensions.

### 5.4. Finite Rendering Robustness and Calibration

Under the confirmatory common long-training budget, F2 retained a small advantage over A3WC in the protocol-defined finite rendering set: +1.69 points in mean seed-wise worst-case balanced accuracy, with a seed-bootstrap 95% interval of +0.45 to +2.78 and exact sign-flip *p* = 0.0352. The effect is materially smaller than the +4.57-point difference observed in the initial six-epoch, five-seed screen, demonstrating sensitivity to the training policy. The nominal K0 difference was −0.26 points and did not exceed the protocol-defined 2-point material-loss threshold, but F2 was worse on average under K2 and failed sharply for the quadcopter and three-long-blade-rotor classes. K2 is a synthetic JPEG transformation, not a new acquisition domain. ResNet18 was the only fully fine-tuned deep backbone, so these results do not demonstrate architecture-independent behavior. The defensible claim is limited to the predefined K0/K1/K3/K4 renderings under paired computational repeats; it does not extend to physical interference, new sensors, acquisition conditions, or other deep architectures.

### 5.5. Limitations and Future Directions

The present study is limited by the form of the publicly available DIAT-μSAT archive. Only rendered JPEG spectrograms were available; raw .mat radar signals, physical amplitude and phase information, and acquisition metadata were not accessible. The new filename-order feature audit was post hoc and establishes only an association between archive index and image representation. It cannot identify the generating process or validate grouping by measurement session, target instance, RPM, aspect angle, or range. S3 is consequently an archive-order extrapolation control, not an acquisition-independent partition. The K1–K4 transformations likewise test image renderings rather than physical SNR or radar-channel changes.

The statistical results are subject to related limitations. The initial policy screen and F0 split comparison use five optimization seeds, whereas the confirmatory long-budget F2-A3WC comparison uses ten paired seeds. Both characterize training variability only within their fixed computational settings and cannot substitute for independent measurement campaigns. At the adopted perceptual-similarity thresholds, S1 and S2 resulted in identical group assignments; therefore, S2 does not constitute an independent or more stringent near-duplicate evaluation. The filename-block bootstrap intervals are conditional on the observed blocks and the trained seed sets used in each analysis; they should not be interpreted as joint uncertainty over training stochasticity or independent acquisition sessions. The dataset also contains only one simultaneous bird-rotor configuration, which prevents broader claims regarding general multi-target recognition.

No independent dataset, acquisition campaign, sensor, site, or physical target population was available for external validation. No experiment varied physical aspect, SNR, distance, RPM, target identity, sensor, or site. All reported controls, including I2C and K0–K4, therefore assess internal sensitivity within the released DIAT-μSAT JPEG archive. They do not establish cross-sensor, cross-site, cross-session, target-population, or physical-condition generalization. Validation on independently acquired raw radar measurements remains necessary before such claims can be made.

The task examined here is radar-based classification of released micro-Doppler spectrogram images; it does not analyze acoustic recordings or distinguish aircraft-engine sounds from bird calls. The archive contains no fixed-wing-airplane class. The label ‘bionic bird’ is retained only as the name of a released archive category and should not be generalized to all natural birds or to aircraft.

Access to the original radar recordings would enable a substantially stronger signal-level evaluation. Future work should therefore include regenerated short-time Fourier transform (STFT) and cadence-velocity representations, radial-velocity normalization, grouping by RPM, aspect angle, and acquisition session, and physically meaningful noise and channel experiments. Ideally, the raw recordings should be accompanied by a mapping between .mat files and the released JPEG images, together with sampling frequency, RPM or flap rate, azimuth, elevation, range, and acquisition-session identifiers. Such metadata would allow direct comparison between image-production shortcuts and genuine signal-level generalization.

## 6. Conclusions

On the released DIAT-μSAT JPEG spectrograms, within-class filename order carries measurable image-feature structure. Adjacent images were more similar than permuted neighbors, and the late S3 test blocks differed from early training blocks more than size-matched random allocations in both frozen ResNet18 and HOG feature spaces. This finding gives the S3 split an observable image-level interpretation without identifying its physical cause. Frozen ResNet18 balanced accuracy was 97.71% under random S0 and 88.64% under S3; the mean five-seed fully fine-tuned F0 gap was 9.94 points. These contrasts show sensitivity to archive-order extrapolation, not session-independent generalization. Duplicate-aware S1 and S2 were identical, and excluding the class with distinct source resolution did not eliminate the frozen-feature gap. Boundary-mask and ring experiments were retained only as non-identifying controls: they cannot separate genuine perimeter time–frequency content from rendering traces or establish shortcut learning.

The second, independent finding concerns finite rendering robustness. Under matched checkpoint selection and a common long-training budget across ten paired seeds, F2 exceeded A3WC by 1.69 points in worst-case balanced accuracy over predefined K0/K1/K3/K4 renderings (seed-bootstrap 95% interval +0.45 to +2.78; exact sign-flip *p* = 0.0352). Nominal K0 performance was approximately retained, but F2 showed no advantage on the held-out K2 stress test. Together, the archive-order audit and paired training comparison support a bounded image-level evaluation framework. Neither establishes generalization across acquisition sessions, sensors, sites, targets, or physical operating conditions; those questions require independent raw measurements and provenance metadata.

## Figures and Tables

**Figure 1:**
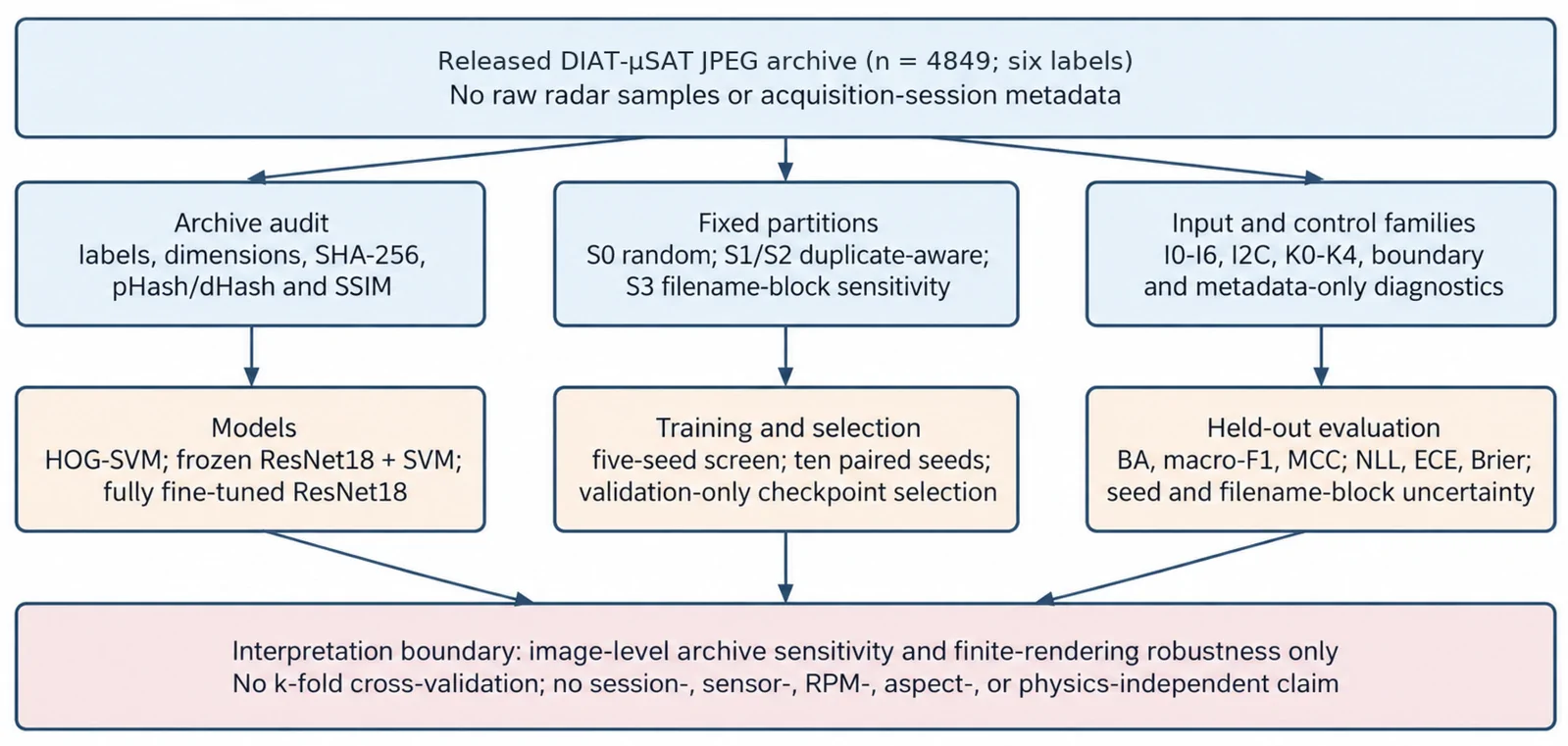
End-to-end computational workflow and claim boundary. The study uses fixed train/validation/test partitions and no k-fold cross-validation. BA: balanced accuracy; ECE: expected calibration error; HOG: histogram of oriented gradients; MCC: Matthews correlation coefficient; NLL: negative log-likelihood; SSIM: structural similarity index; SVM: support vector machine.

**Figure 2:**
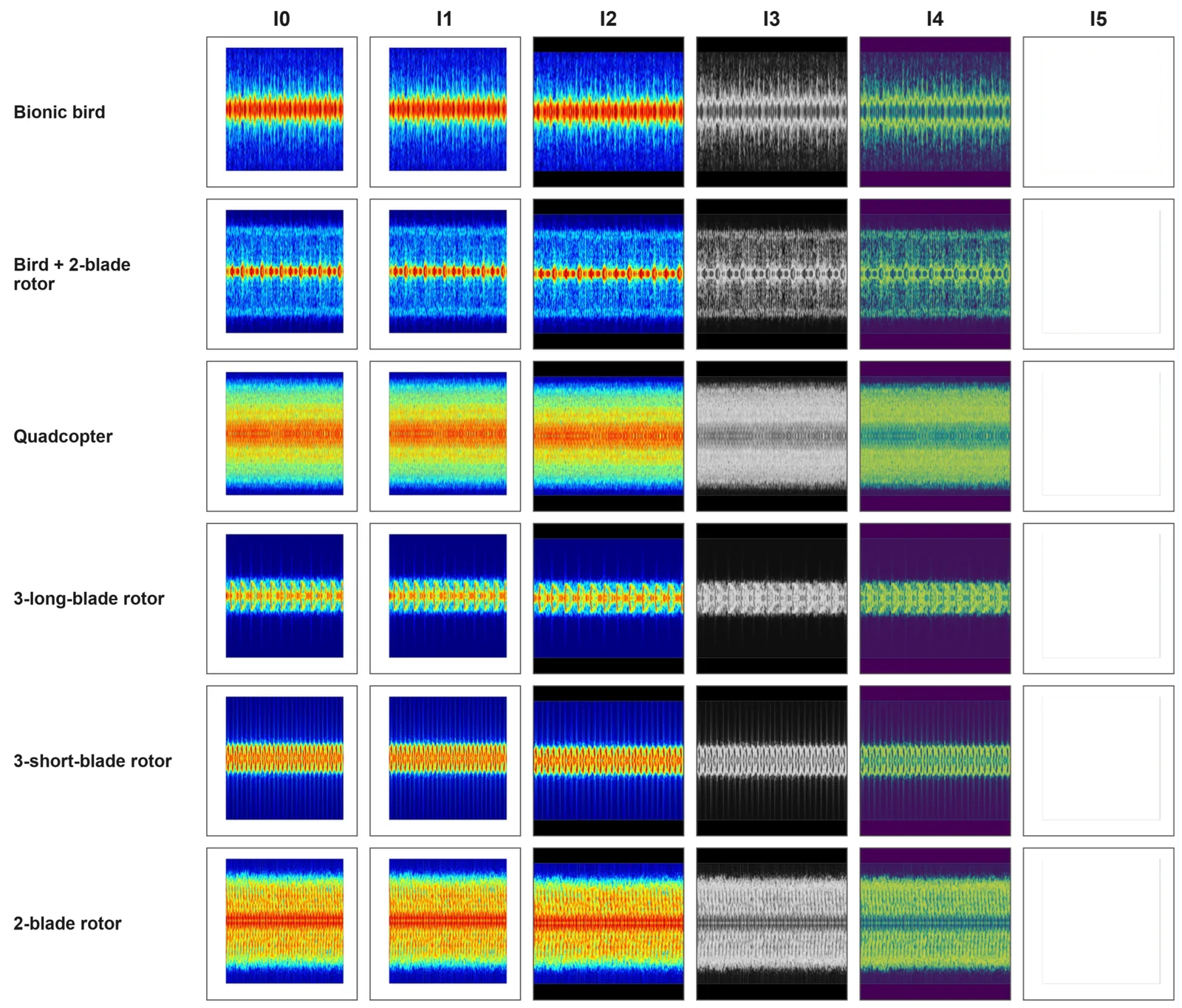
Visual contact sheet for the I0–I5 image-input variants. I2C is the common-intermediate-resolution sensitivity control, and I6 is a non-image metadata control. The displayed colors are the actual pixel renderings of the image variants rather than categorical annotations. The I2 red-green-blue (RGB) spectrogram region of interest (ROI), which removes only the surrounding white canvas, was selected as the primary image input.

**Figure 3:**
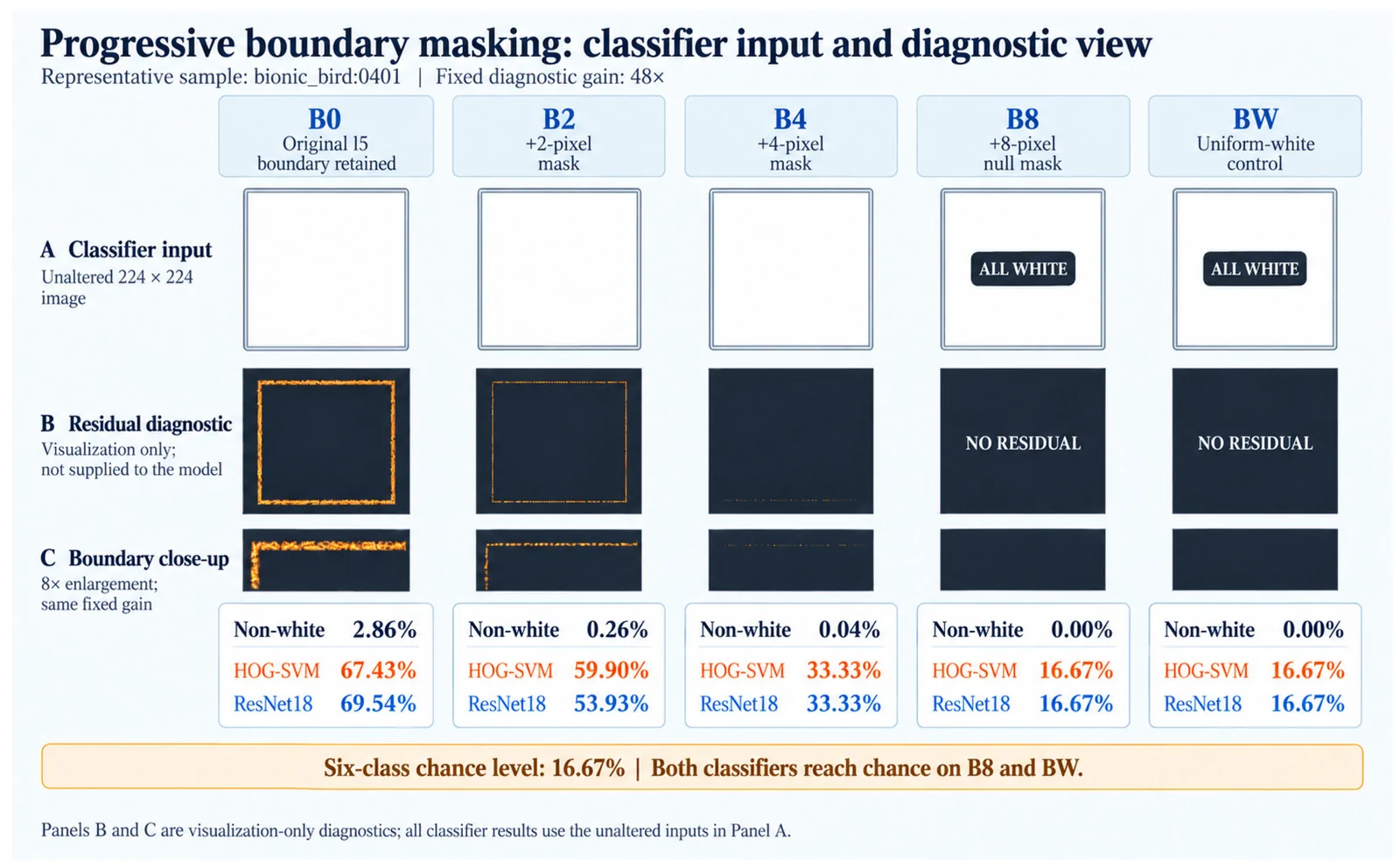
Effect of progressive boundary masking for a representative bionic-bird sample. Panel (**A**) shows the actual 224 × 224 classifier inputs. Panels (**B**) and (**C**) are fixed-gain diagnostic views of the residual maps and an enlarged upper ROI boundary; they were not supplied to the classifiers. The outlined boxes separate the classifier inputs, diagnostic views, and summary metrics; orange labels denote HOG-SVM results, whereas blue labels denote ResNet18 results. Non-white boundary residuals decrease from B0 to B4, whereas B8 and BW are uniformly white. The retained pixels may include both the perimeter of genuine time–frequency content and rendering traces; the image-only experiment cannot separate these origins. The lower strip reports S3 balanced accuracy and non-white pixel proportions.

**Figure 4:**
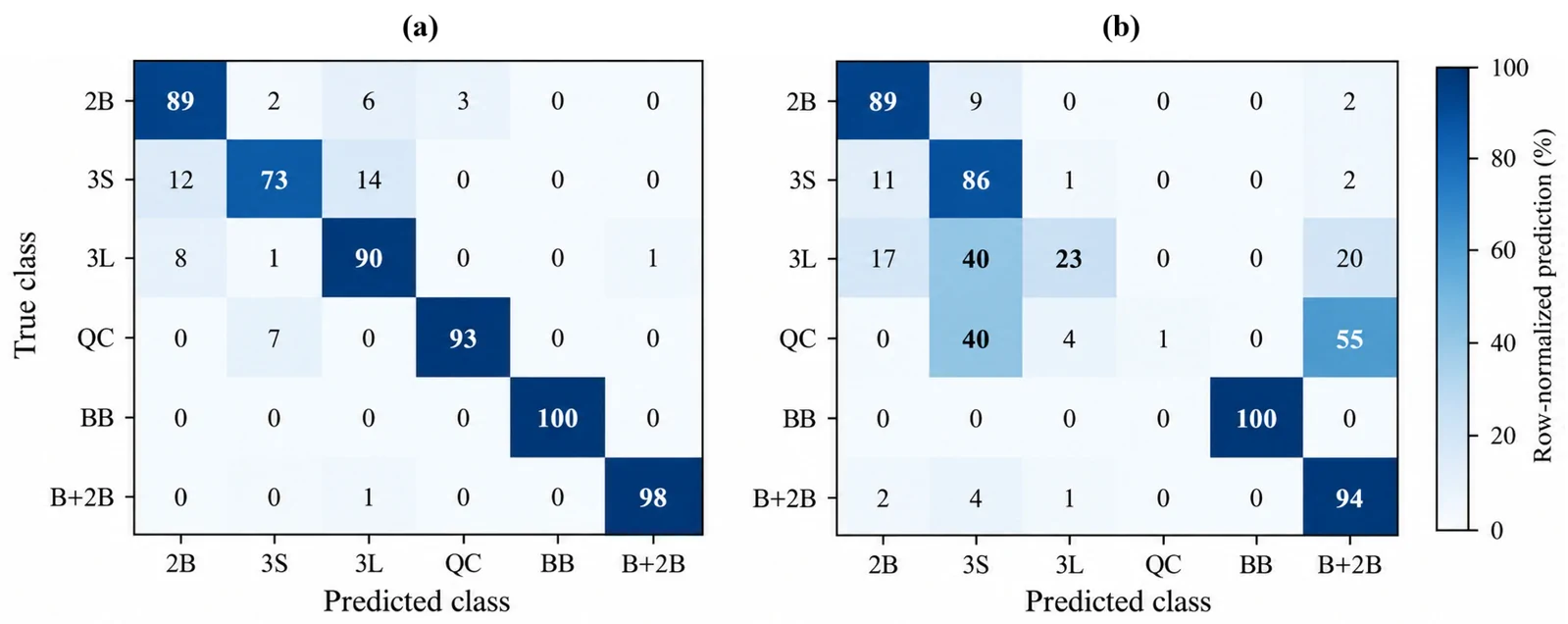
Mean row-normalized F2 confusion matrices across the ten paired optimization seeds: (**a**) original K0 RGB rendering; (**b**) synthetic K2 histogram-equalized stress rendering. Rows denote true classes, and columns denote predicted classes; every row sums to 100%, and each cell reports the across-seed mean percentage, rounded to the nearest integer, of that true class assigned to the predicted class. Bold values denote diagonal entries (correct-class recall). K2 was excluded from training and checkpoint selection. Under K2, the largest recall losses occur for the three-long-blade-rotor and quadcopter classes.

**Table 1:** Principal findings of the archive audit.

Item	Finding	Academic Implication
Total samples	4849 JPEGs/6 classes	Raw radar measurements are unavailable.
Class counts	800, 815, 835, 799, 800, 800	The classes are nearly balanced.
Source resolution	Bionic bird: 700 × 525; others: 1400 × 1050	Class-associated rendering factor; evaluated with the I2C common-intermediate-resolution control.
Exact duplicates	2 pairs	Members are kept in the same partition.
Missing index	Three-long-blade rotor: 354	Filename order is not a session identifier.

**Table 2:** Sample counts and roles of the fixed data partitions.

Partition	Training	Validation	Test	Role
S0 random	3879	486	484	Secondary comparison with literature
S1 duplicate-grouped	3879	486	484	Exact-duplicate control
S2 similarity-grouped	3879	486	484	Audit-only; identical to S1, not separate evidence
S3 filename-blocked	3899	480	470	Main filename-order sensitivity partition

**Table 3:** Input family and experimental roles.

Code	Content	Role
I0	Original full RGB JPEG	Published-image reference
I1	Commonly recoded full image	Source-rendering control
I2	RGB spectrogram ROI without outer white canvas	Primary image input
I2C	I2 ROI with common 542 × 428 intermediate pixel resolution	Source-resolution sensitivity control
I3	Grayscale ROI	Color-dependence control
I4	Alternative contrast/color mapping	Mapping ablation
I5	Spectrogram-masked image	Boundary-residual diagnostic
I6	File/JPEG attributes	Metadata-only archive diagnostic

**Table 4:** S0–S3 partition chain for frozen ResNet18-I2. BA is balanced accuracy; MCC is the Matthews correlation coefficient.

Partition	Balanced Accuracy	Macro-F1	MCC
S0 random	97.71%	97.70%	0.973
S1 duplicate-grouped	96.67%	96.67%	0.960
S2 similarity-grouped	96.67%	96.67%	0.960
S3 filename-blocked	88.64%	88.50%	0.863

**Table 5:** Metadata-only decision-tree ablation on S3.

Metadata Input	Tree Depth	Balanced Accuracy	Macro-F1
Width + height	1	33.33%	22.04%
File size only	2	44.38%	33.01%
JPEG fields only	1	16.67%	4.59%
All metadata	2	44.38%	33.01%

**Table 6:** Confirmatory long-budget ResNet18 comparison over ten paired seeds. Values are mean ± sample standard deviation.

Model	K0	K1	K2	K3	K4	Allowed-Set Worst
A3WC matched-select (long *n* = 10)	90.86 ± 1.20	89.73 ± 1.47	68.18 ± 3.17	88.55 ± 1.30	89.18 ± 1.73	88.25 ± 1.28
F2 randomized (long *n* = 10)	90.60 ± 1.83	90.48 ± 1.92	65.36 ± 2.81	91.19 ± 1.07	92.59 ± 0.96	89.94 ± 1.57

**Table 7:** Reported results and validation limits in selected DIAT-μSAT studies.

Study	Input and Reported Result	Validation and Evidence Limit
Kumawat et al. [19]	4849 DIAT JPEGs; VGG16/19: 95%/97%.	Random 80/10/10 image split; no duplicate or acquisition-group control reported; no external validation.
Kumawat et al. [21]	DIAT spectrograms; DIAT-RadSATNet: 97.1–97.3%.	Held-out ‘unknown’ DIAT images; grouping unit undisclosed; no archive-level dependence audit or independent dataset.
Hasan et al. [22]	4860 range- and micro-Doppler images; R-PCA + SVM: 98%.	Internal test; grouping unit unreported; no duplicate or batch control or external validation reported.
Agrebi and Neyt [23]	Four-class temporal micro-Doppler signals; GRU: >98%.	Internal random split; no acquisition-group control or external validation reported.
Agrebi et al. [24]	Four-class temporal signals with synthetic AWGN; GRU: F1 > 0.9.	Internal clean/noisy evaluation; not a duplicate/batch audit; no measured external sensor or SNR dataset.
Libal et al. [25]	Six classes, 108 spectral features; IQGA-SVM: 99.59%.	Internal random partition; no acquisition-group control or external validation reported.
Chakraborty et al. [26,27]	DIAT frequency-domain complex inputs; complex CNN: about 99%.	Internal DIAT test; no archive-level dependence audit or independent dataset reported.
Paul and Bandyopadhyay [29]	DIAT JPEGs and RRN_ATR_NET; attentive binarized CNN: 97.83% DIAT, 99.17% RRN.	Dataset-specific validation; DIAT acquisition groups unreported. A second dataset was tested, but DIAT shortcut factors were not.
This study	4849 DIAT JPEGs; fixed ROI and I2C control; ResNet18 F2 vs. A3WC: +1.69 pp.	S0-S3; ten paired seeds; matched checkpoint selection; duplicate, filename-order, file-attribute, and rendering controls. No independent acquisition.

## Data Availability

The original DIAT-μSAT JPEG images are available through the dataset record cited in this article and are not redistributed here. The accompanying archive supports image-level computational reproduction of the reported audit, partitions, transformations, model analyses, and manuscript statistics. It does not reproduce radar acquisition, waveform generation, analog/front-end processing, or spectrogram formation from raw measurements; consequently, it does not provide experimental reproducibility, and computational reproducibility does not compensate for unavailable raw measurement evidence. The archive contains the audit manifest, S0–S3 and sensitivity split files, duplicate lists, ROI and rendering configurations, training and matched-selection configurations, seed lists, analysis and bootstrap scripts, per-sample predictions, and machine-readable result summaries. Pretrained weights are not redistributed; the specified initialization is torchvision ResNet18_Weights.IMAGENET1K_V1, corresponding to resnet18-f37072fd.pth with SHA-256 f37072fd47e89c5e827621c5baffa7500819f7896bbacec160b1a16c560e07ec. The revision package additionally contains the locked ten-seed long-budget configuration, all 100 held-out prediction files, compact statistical summaries, and a regenerated SHA-256 inventory. The implementation used torchvision 0.28.0+cpu, distributed under the BSD 3-Clause software license (https://github.com/pytorch/vision/blob/main/LICENSE (accessed on 19 September 2026)).

## Supplementary Materials

The following supporting information can be downloaded at: https://www.mdpi.com/article/10.3390/s26195989/s1, Figure S1: Balanced accuracy across the seven filename-block sensitivity policies; Table S1: Sensitivity of the filename-blocked evaluation to block length and grid offset; Table S2: Seed-wise confirmatory long-budget F2-A3WC results (balanced-accuracy differences in percentage points); Table S3: Compact confirmatory uncertainty and stress-test summary; Table S4: Mean calibration metrics before (U0) and after (C1) temperature scaling; Table S5a: Software environment used for the confirmatory long-budget analysis; Table S5b: Contents of the reproducibility package; Table S6: Boundary-ring and edge-suppression results on S3; Table S7: Five-class sensitivity results after exclusion of the bionic-bird category; Table S8: Exploratory within-class archive-order structure in two I2 feature families. Sections S1–S8 provide the corresponding methods, limitations, and interpretations. The accompanying reproducibility package contains audit manifests, fixed and sensitivity splits, configurations, scripts, per-sample predictions, and machine-readable summaries. The original DIAT-μSAT JPEG archive and pretrained ImageNet weights are not redistributed.

## Abbreviations

BA, balanced accuracy; CNN, convolutional neural network; DCT, discrete cosine transform; ECE, expected calibration error; HOG, histogram of oriented gradients; JPEG, Joint Photographic Experts Group; MCC, Matthews correlation coefficient; NLL, negative log-likelihood; pHash/dHash, perceptual/difference hash; RGB, red-green-blue; ROI, region of interest; RPM, revolutions per minute; SNR, signal-to-noise ratio; SSIM, structural similarity index; STFT, short-time Fourier transform; SVM, support vector machine.
