# Supplementary material for: Assessing the Reliability of Micro-Doppler Spectrogram Classification from the DIAT-μSAT JPEG Archive
Source: Sensors (Basel). 2026 Sep 22;26(19):5989. doi: 10.3390/s26195989 (PMC13667288; doi:10.3390/s26195989)
Supplement: Supplementary file 1 [file sensors-26-05989-s001.zip › sensors-4533178-supplementary.pdf]

Supplementary Material

Assessing the Reliability of Micro-Doppler Spectrogram Classification from the DIAT-μSAT JPEG Archive

Mert Karahan 1 and Şevki Gani Şanlıöz 2,*

1 Department of Electronics and Communication Engineering, Turkish Military Academy, National Defence University, 06420 Ankara, Türkiye; mert.karahan@msu.edu.tr

2 Faculty of Engineering and Natural Sciences, Istanbul Atlas University, 34408 Istanbul, Türkiye

* Correspondence: gani.sanlioz@atlas.edu.tr

S1. Filename-block sensitivity analysis

The main S3 diagnostic partition uses class-stratified contiguous filename blocks of length 20. To test dependence on this construction choice, seven variants were generated with block lengths 10, 20, 40, and 80 and, for length 20, grid offsets 0, 5, 10, and 15. A short tail containing fewer than half the configured block length was merged into the preceding block. Hyperparameters were selected on the corresponding validation partition. These overlapping held-out sets provide a descriptive protocol-sensitivity analysis and are not independent acquisitions, session labels, or proof of a non-i.i.d. physical split.

Policy	Block length	Offset	Test n	HOG-SVM BA (%)	Frozen ResNet18 BA (%)
S3_L010_O000	10	0	480	81.30	88.74
S3_L020_O000	20	0	470	81.18	88.64
S3_L020_O005	20	5	500	81.68	88.73
S3_L020_O010	20	10	450	81.05	90.10
S3_L020_O015	20	15	460	81.28	89.86
S3_L040_O000	40	0	490	79.73	88.91
S3_L080_O000	80	0	530	79.39	88.64

Table S1. Sensitivity of the filename-blocked evaluation to block length and grid offset.

Across all variants, HOG-SVM balanced accuracy ranged from 79.39% to 81.68%, and frozen-ResNet18 balanced accuracy ranged from 88.64% to 90.10%. At zero offset, changing block length produced only 88.64–88.91% for frozen ResNet18; varying the L20 origin produced 88.64–90.10%. The random S0 reference remained 97.71%, so the principal random-to-blocked reduction was larger than the observed construction sensitivity.

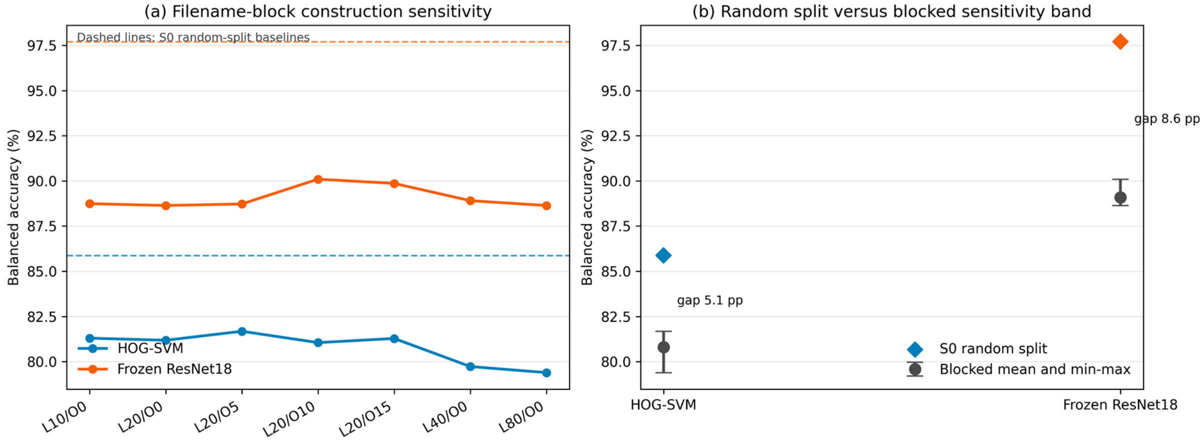

Figure S1. Balanced accuracy across the seven filename-block sensitivity policies.

S2. Confirmatory paired-seed results

Table S2 reports every paired seed from the confirmatory common-long-budget F2-A3WC experiment. Both policies used identical S3 identities, optimization seeds, epoch and early-stopping budgets, and worst-case K0/K1/K3/K4 checkpoint selection. The table is provided to expose the computational-repeat structure behind the mean effect; it does not represent independent radar acquisitions.

Seed	F2 worst	A3WC worst	Δ worst	Δ K0	Δ K2
2026	91.24	88.93	+2.31	+0.65	-2.67
2027	89.46	89.47	-0.01	+1.67	-3.46
2028	87.35	88.85	-1.50	-5.00	-0.76
2029	88.90	86.40	+2.50	-1.25	+3.38
2030	90.51	87.21	+3.31	-0.21	-3.36
2031	90.08	87.35	+2.74	+0.72	-0.54
2032	90.12	87.39	+2.74	+1.40	-2.79
2033	88.17	89.81	-1.64	-1.94	-8.76
2034	92.79	89.94	+2.85	+0.82	-7.17
2035	90.82	87.17	+3.65	+0.51	-2.07

Table S2. Seed-wise confirmatory long-budget F2-A3WC results (balanced-accuracy differences in percentage points).

F2 exceeded A3WC on the protocol-defined allowed-set worst-case endpoint in seven of ten seed pairs and was lower in three. The mean paired difference was +1.69 percentage points; K0 and K2 columns are secondary descriptions.

S3. Compact uncertainty and stress-test summary

The primary uncertainty analysis resampled the ten paired optimization-seed differences. A complementary analysis resampled the 24 observed filename blocks within class while holding trained models fixed. The two intervals answer different conditional questions and neither captures variation across acquisition sessions, sensors, targets, or physical operating conditions.

Quantity	Estimate	Seed 95% interval	Block 95% interval	Status
F2−A3WC allowed-set worst case	+1.69 pp	+0.45 to +2.78	+0.74 to +3.56	Primary; p=0.0352
F2−A3WC K0	−0.26 pp	−1.55 to +0.79	—	Secondary
F2−A3WC K2	−2.82 pp	—	—	Secondary synthetic stress
Direction across seed pairs	7 positive; 3 negative	—	—	Computational repeats

Table S3. Compact confirmatory uncertainty and stress-test summary.

Only the allowed-set worst-case comparison was protocol-defined as the primary endpoint. K0 and K2 results are secondary; K2 is a synthetic histogram-equalized JPEG rendering and is not evidence of a physical radar-domain shift.

S4. Compact calibration summary

Scalar temperature scaling C1 was fitted without K2 and did not change predicted class labels. Metrics below are means over the allowed K0/K1/K3/K4 rendering set; K2 NLL is shown only for F2 as a secondary descriptive stress result.

Table S4. Mean calibration metrics before (U0) and after (C1) temperature scaling.

Model	State	NLL	ECE	Brier	K2 NLL
F0	U0	0.6289	0.0714	0.3170	—
F0	C1	0.6412	0.0788	0.3207	—
F2	U0	0.3210	0.0578	0.1592	1.4803
F2	C1	0.3009	0.0496	0.1553	1.3567

C1 did not improve F0 calibration under the predefined descriptive criteria. For F2, C1 reduced the three allowed-set metrics; the seed-paired 95% interval for the C1−U0 NLL difference was [−0.0347, −0.0055].

S5. Reproducibility package and software environment

S5.1 Software and execution environment

The confirmatory long-budget experiment was executed on CPU with CUDA disabled, eight computational threads, and zero data-loader workers. The versions below were read from the execution environment used for the run; the complete environment specification is included as requirements_revision1_e1.txt.

Table S5a. Software environment used for the confirmatory long-budget analysis.

Component	Version or setting	Role
Python	3.12.10	Execution runtime
PyTorch	2.13.0+cpu	Training and inference
torchvision	0.28.0+cpu	ResNet18 model and image transforms
NumPy	2.5.1	Numerical arrays and resampling
SciPy	1.18.0	Numerical optimization
pandas	3.0.5	Manifests and result tables
scikit-learn	1.9.0	SVMs, metrics, and metadata diagnostic
scikit-image	0.26.0	Image similarity calculations
Pillow	12.3.0	JPEG decoding and deterministic rendering
matplotlib	3.11.1	Scientific plots
Execution	CPU; CUDA disabled; 8 threads; 0 workers	Paired deterministic long-budget runs

S5.2 Package contents

The accompanying ZIP supports image-level reproduction of the audit, splits, transformations, models, and statistics. It redistributes neither the DIAT-μSAT JPEG archive nor ImageNet weights and cannot reproduce radar acquisition or spectrogram formation from raw measurements. Computational reproduction cannot replace unavailable experimental evidence. Initialization uses torchvision ResNet18_Weights.IMAGENET1K_V1 (resnet18-f37072fd.pth; SHA-256 f37072fd47e89c5e827621c5baffa7500819f7896bbacec160b1a16c560e07ec). The first revision added the locked ten-seed configuration, 100 held-out prediction files (two models × ten seeds × five renderings), and compact E1 summaries. This revision adds scripts/archive_order_audit.py, results/archive_order_audit.json, ARCHIVE_ORDER_AUDIT.md, and a regenerated SHA-256 inventory. torchvision 0.28.0+cpu is BSD-3-Clause licensed; its source and pretrained weights are not redistributed.

Package component	Contents
audit/	Manifest, duplicate lists, S0–S3 splits, and block-sensitivity splits
config/	ROI, rendering, training, calibration, and pilot JSON configurations
scripts/	Audit, transform, training, calibration, bootstrap, and summary scripts
results/	Prediction CSV files and machine-readable CSV/JSON/Markdown summaries
PACKAGE_MANIFEST_SHA256.csv	Relative paths, byte sizes, and SHA-256 checksums for package files
requirements.txt	Pinned Python dependencies used by modelling, audit, figure, and report scripts
matched-selection control	paired-seed comparison, uncertainty, and calibration summaries
config/revision1_e1_long_training.json	Locked ten-seed F2–A3WC long-budget protocol.
results/revision1_e1_long_training/	All result JSON and 100 held-out prediction CSV files; checkpoints excluded.
results/revision1_e1_analysis/	Paired seed, representation, class-recall, and bootstrap summaries.
requirements_revision1_e1.txt	Exact core package versions from the long-budget execution environment.

Table S5b. Contents of the reproducibility package.

S6. Boundary-ring and edge-suppression controls

These controls use the fixed S3 identities. C0 is the unmodified I2 ROI; C2, C4, and C8 suppress progressively wider strips along the inner content boundary. R2, R4, and R8 retain only isolated boundary rings after masking the central spectrogram region. Ring-only performance remains above the six-class chance level of 16.67%, but the rings also preserve the perimeter of genuine time-frequency content. The controls therefore show that boundary-restricted inputs are class-informative without identifying the retained information as a non-physical rendering artifact. The non-monotonic edge-suppression results likewise do not support an explanation based solely on a few boundary pixels.

Table S6. Boundary-ring and edge-suppression results on S3.

Control	Description	Non-background fraction	HOG-SVM BA (%)	ResNet18 BA (%)
C0	Unmodified I2 ROI	79.02%	81.18	88.64
C2	Suppress 2-pixel inner boundary	79.02%	79.50	86.97
C4	Suppress 4-pixel inner boundary	79.02%	77.85	88.63
C8	Suppress 8-pixel inner boundary	79.02%	82.33	90.76
R2	Isolated 2-pixel boundary ring	3.16%	67.12	75.03
R4	Isolated 4-pixel boundary ring	6.27%	68.44	78.61
R8	Isolated 8-pixel boundary ring	12.28%	73.74	81.97

S7. Source-resolution class-exclusion sensitivity control

This additional reviewer-motivated sensitivity analysis tested whether the S0–S3 reduction was driven solely by the bionic-bird category, the only class with a different native source-image resolution. All 800 bionic-bird images were excluded; the I2 inputs and the S0/S3 memberships of the remaining 4,049 images were unchanged. The original validation grids and tie-breaking rules were retained, the LinearSVC hyperparameter was reselected on each filtered validation set, and the classifier head was refitted on filtered train plus validation data. The S0 and S3 test sets contained 404 and 390 images, respectively.

Table S7. Five-class sensitivity results after exclusion of the bionic-bird category.

Model	S0 BA (%)	S3 BA (%)	S0-S3 gap (pp)	95% interval (pp)
HOG-SVM	83.30	78.18	+5.12	-0.16 to +10.33
Frozen ResNet18 + LinearSVM	97.25	86.12	+11.13	+7.76 to +14.87

For HOG-SVM, validation selected C = 0.01 on S0 and C = 0.1 on S3; the corresponding macro-F1/MCC values were 83.18%/0.793 and 78.03%/0.726. For frozen ResNet18 features, validation selected C = 1.0 on S0 and C = 0.1 on S3; macro-F1/MCC values were 97.24%/0.966 and 86.00%/0.825. Intervals were computed from 5,000 independent class-stratified resamples of each protocol's test set (seed 20260910). Because the test identities differ between S0 and S3, the intervals are diagnostic sensitivity summaries rather than paired or session-independent estimates.

S8. Exploratory image-feature structure along filename order

This post-hoc audit used cached I2 HOG and ImageNet-frozen ResNet18 features from all 4,849 JPEG images. Features were L2-normalized within their native feature spaces. For each published class, we tested whether consecutive filename indices had higher mean cosine similarity than 499 random permutations of the within-class order. We also compared the centroid distance between S3 early training and late test samples with 499 random within-class allocations preserving the observed train and test counts; validation samples did not enter the centroid statistic. The empirical one-sided p-value was (1 + number of permutation statistics at least as large as observed)/500. These comparisons were conceived after the first revision and are exploratory, not a physical-session validation.

Class	ResNet adjacent excess	HOG adjacent excess	ResNet late-distance ratio	HOG late-distance ratio
bionic bird	0.01975	0.00456	2.25	1.56
bird plus two blade rotor	0.06400	0.05245	2.35	2.40
quadcopter	0.02613	0.02430	4.13	4.53
three long blade rotor	0.04827	0.04093	2.43	3.31
three short blade rotor	0.07025	0.06459	4.87	3.51
two blade rotor	0.07504	0.06919	4.53	2.83

Table S8. Exploratory within-class archive-order structure in two I2 feature families. Adjacent excess is the observed mean consecutive-pair cosine similarity minus the mean of 499 permuted orders. Late-distance ratio is the observed S3 train–test centroid distance divided by the mean distance under 499 size-matched random assignments. All class-specific empirical one-sided p-values were 0.002, the minimum resolvable value with 499 permutations; they are descriptive post-hoc diagnostics rather than a prespecified inferential family.

The same direction held for all six classes when the two tests were repeated on grayscale I3 features after removing one image from each exact-duplicate pair: ResNet18 adjacent excess 0.02416–0.09998 and late-distance ratios 1.95–5.64; HOG adjacent excess 0.00437–0.06736 and late-distance ratios 1.53–4.59. The source of the ordering remains unverified. These results cannot convert filename blocks into acquisition-session or physical-domain groups.
